# The effects of polyphenol supplementation on adipose tissue morphology and gene expression in overweight and obese humans

**DOI:** 10.1080/21623945.2018.1469942

**Published:** 2018-05-22

**Authors:** Jasper Most, Ines Warnke, Mark V. Boekschoten, Johan W. E. Jocken, Philip de Groot, Angelika Friedel, Igor Bendik, Gijs H. Goossens, Ellen E. Blaak

**Affiliations:** a Department of Human Biology, NUTRIM School of Nutrition and Translational Research in Metabolism, Maastricht University Medical Center+, the Netherlands; b DSM Nutritional Products Ltd., Research and Development, Human Nutrition and Health; Basel, Switzerland; c Nutrition, Metabolism and Genomics Group, Division of Human Nutrition, Wageningen University, Wageningen, The Netherlands

**Keywords:** Adipose tissue, EGCG, Morphology, Obesity, Resveratrol, Transcriptomics

## Abstract

Dietary polyphenols have beneficial effects on adipose tissue mass and function in rodents, but human studies are scarce. In a randomized, placebo-controlled study, 25 (10 women) overweight and obese humans received a combination of the polyphenols epigallocatechin-gallate and resveratrol (282 mg/d, 80 mg/d, respectively, EGCG+RES, n = 11) or placebo (PLA, n = 14) supplementation for 12 weeks. Abdominal subcutaneous adipose tissue (SAT) biopsies were collected for assessment of adipocyte morphology and micro-array analysis. EGCG+RES had no effects on adipocyte size and distribution compared with PLA. However, we identified pathways contributing to adipogenesis, cell cycle and apoptosis were significantly downregulated by EGCG+RES *versus* PLA. Furthermore, EGCG+RES significantly decreased expression of pathways related to energy metabolism, oxidative stress, inflammation, and immune defense as compared with PLA. In conclusion, the SAT gene expression profile indicates a reduced cell turnover after 12-week EGCG+RES in overweight-obese subjects. It remains to be elucidated whether these alterations translate into long-term metabolic effects.

## Introduction

Enlargement of abdominal subcutaneous adipocytes is an independent marker of insulin resistance [1], and predicts the development of type 2 diabetes [2]. Additionally, adipose tissue dysfunction rather than an increased fat mass *per se* is associated with the development of insulin resistance. This suggests that both the morphology and function of adipose tissue (AT) determine the risk of developing chronic metabolic conditions accompanying the obese phenotype.

Epigallocatechin-3-gallate (EGCG) and resveratrol (RES) are dietary polyphenols, abundantly available in green tea and in grapes, respectively. Both have been shown to prevent the development of fat mass accretion and insulin resistance in rodents on obesogenic diets via inhibition of adipogenesis and inflammation, and increased lipolysis and energy expenditure [3–6]. However, most human studies have not found significant effects on AT mass and whole-body metabolic profile after supplementation with either EGCG or RES [7,8]. Nevertheless, RES supplementation for 4 weeks induced a reduction in adipocyte size in obese men [9], which was accompanied by an AT gene expression profile indicative of increased adipose tissue turnover including adipogenesis, autophagy and inflammation.

We have recently postulated that combining different polyphenols may lead to additional and/or synergistic and, therefore, more pronounced metabolic effects compared with single supplementation. Indeed, while EGCG supplementation alone for 3 days was ineffective [10], combined EGCG and RES (EGCG+RES) supplementation increased energy expenditure and plasma leptin concentrations in overweight subjects [11]. More recently, we have shown that combined EGCG+RES supplementation for 12 weeks increased whole-body fat oxidation and mitochondrial capacity in skeletal muscle, but did not significantly alter whole-body lipolysis, AT and skeletal muscle lipolysis and tissue-specific insulin sensitivity [12]. Here, we investigated whether EGCG+RES supplementation for 12 weeks induces alterations in abdominal subcutaneous AT morphology and gene expression profiles in overweight and obese men and women compared with placebo (PLA).

## Results

### Subjects characteristics

Twenty-five subjects (10 women, 15 men) were included in the present sub-study, since AT biopsies were not available for all individuals that completed the main trial (n = 38). At baseline, subject characteristics and sex-distribution were not significantly different between groups in the total study population [12], and were comparable in the present sub-study (14 PLA; 8 men, 6 women; 11 EGCG+RES, 7 men, 4 women, Table 1).

**Table 1.** Subject characteristics and plasma biochemistry.

	PLA, n = 14		EGCG+RES, n = 11		
	Week 0	Week 12	Week 0	Week 12	P
Age, years	40±3		36±3		
BMI, kg/m^2^	29.7±1.1		30.5±0.7		
Waist-to-hip-ratio	0.88±0.03		0.88±0.03		
Diastolic BP, mmHg	111±3		119±2		
Systolic BP, mmHg	74±2		76±2		
HbA1c, %	5.15±0.08		5.05±0.06		
Glucose, mmol/l	5.11±0.11	5.12±0.12	5.13±0.13	5.08±0.14	0.50
2h-Glucose, mmol/l	5.29±0.29		5.19±0.3		
Insulin, mU/l	9.0±1.2	10.6±1.1	8.7±1.3	7.8±1.2	0.03
HOMA-IR	2.03±0.26	2.39±0.24	1.97±0.29	1.77±0.27	0.03
Free fatty acids, µmol/l	532±32	491±18	497±55	532±53	0.33
Triacylglycerol, mmol/l	1.06±0.22	1.31±0.24	1.75±0.25	1.93±0.27	0.65
Adiponectin, µg/ml	8.3±1.0	8.6±1.0	7.0±1.1	7.1±1.1	0.77
Leptin, ng/ml	23.1±4.5	24.3±4.5	17.8±5.1	16.1±5.1	0.16
Interleukin-6, pg/ml	1.00±0.17	0.91±0.13	0.69±0.19	0.77±0.14	0.46
Interleukin-8, pg/ml	9.28±0.94	10.32±1.06	9.79±1.07	9.79±1.20	0.43
TNF-α, pg/ml	2.86±0.24	3.3±0.46	2.87±0.27	2.81±0.52	0.26
EGCG, ng/ml	0±0	0±0	0±0	15±10	<0.01
RES, ng/ml	0±0	0±0	0±0	233±55	<0.01
Dihydro-RES, ng/ml	0±0	0±0	0±0	177±35	<0.01

BMI, Body-Mass-Index; BP, blood pressure; HbA1c, glycated hemoglobin A 1c; 2h-glucose, plasma glucose after oral glucose-tolerance test; HOMA-IR, Homeostatic Model Assessment of insulin resistance; TNF-α, tumor necrosis factor alpha; EGCG, epigallocatechin-3-gallate; RES, resveratrol. Values given as mean±SEM. P, P-value for statistical significance of time*treatment interaction.

### Adipocyte size

**Figure 1.**
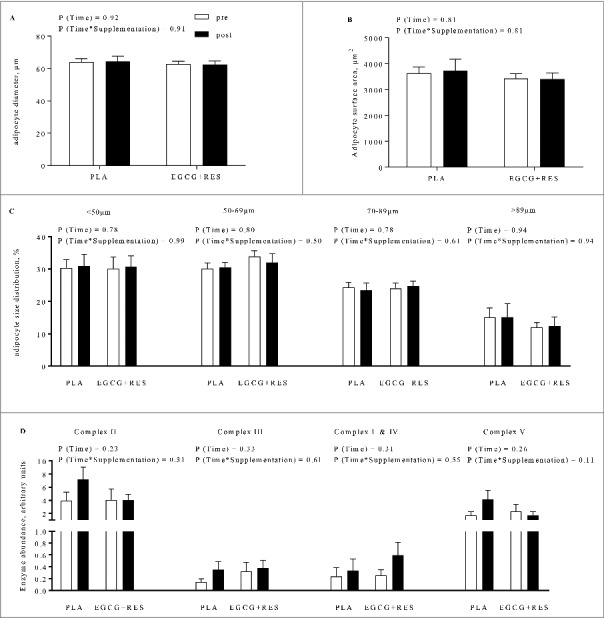
Abdominal subcutaneous adipocyte morphology and OxPhos protein expression before and after intervention. A) Mean adipocyte diameter, B) surface area, and C) adipocyte size distribution, and D) mitochondrial protein content of complexes I-V (complex II: succinate-dehydrogenase, III: ubiquinol-cytochrome C reductase, I&IV: NADH-dehydrogenase & cytochrome C oxidase, and V: ATP-synthase) were not affected by EGCG+RES supplementation compared with PLA. Open bars: week 0; solid bars: week 12. Statistical significance of time and time*supplementation interaction is indicated as P. Values are given as means ± SEM (EGCG+RES, n = 5; PLA n = 9).

EGCG+RES supplementation had no significant effect on mean adipocyte size or surface area in abdominal subcutaneous AT (Figure 1A-B). In line, adipocyte size distribution was unchanged after EGCG+RES supplementation (Figure 1C), indicating that EGCG+RES did not induce a shift from large to small adipocytes or vice versa.

### Adipose tissue gene expression profile

Of the 26876 genes on the array, 10987 were analyzed after filtering (Interquartile range>0.2 (log2), intensity>20 on at least 5 arrays, >5 probes/gene). 763 genes were differentially expressed after EGCG+RES supplementation as compared to PLA (P<0.05), of which 424 genes showed higher expression levels (Supplementary Table S1).

**Figure 2.**
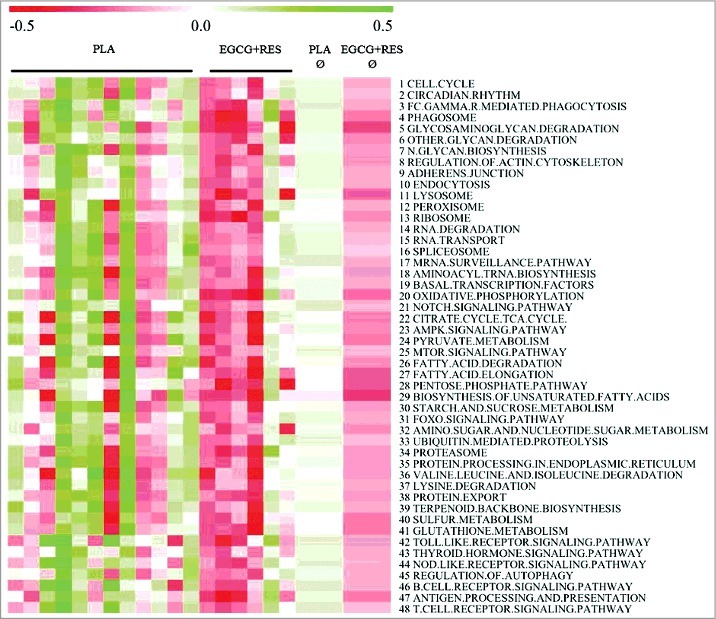
Adipose tissue gene expression changes by intervention. Gene set enrichment analysis (GSEA) of adipose tissue micro-array data revealed that gene sets (Kyoto Encyclopedia of Genes, Genomes, Wikipathways, and Biocarta database) related to cell turnover (1-19), energy and substrate metabolism (20-40), inflammation and the immune system (41-48) were significantly downregulated following EGCG+RES supplementation (PLA, n = 12; EGCG+RES, n = 6, data are first presented by individual and in the last two columns as average per group). Red color indicates downregulated pathways, whereas green color indicates upregulated pathways after EGCG+RES *versus* PLA. Functional data analysis was based upon FDR q-value <0.05 on the filtered data set (interquartile range > 0.2 (log2), intensity >20 on at least 5 arrays, >5 probes per gene) for the interaction (EGCG+RES versus PLA) with GSEA which was run with 1000 permutations.

Using the Databases Kyoto Encyclopedia of Genes and Genomes, Wikipathways, and Biocarta, we identified 332 pathways that were differentially expressed between EGCG+RES and PLA. Strikingly, expression levels of all these pathways were lower after EGCG+RES supplementation as compared to the PLA group, as indicated by negative normalized enrichment scores, whereas no pathways were significantly enriched after EGCC+RES supplementation (Supplementary Table S2). More specific, gene sets related to cell turnover (circadian rhythm, cytoskeleton and apoptosis/autophagy) and transcription and translation showed lower expression levels (Figure 2, rows 1–19). Furthermore, EGCG+RES decreased the expression of pathways related to energy and substrate metabolism, oxidative stress, immune defense (Figure 2, rows 20–48) and various diseases, including Alzheimer's, several types of cancer, and infectious, immune, and inflammatory diseases (Supplementary Table S2). Next, we selected individual genes – by significance (P<0.05) and fold-change (FC>1.25) in the micro-array analysis – and measured their mRNA expression using quantitative real-time PCR (RT-qPCR), with GAPDH as housekeeping gene. We observed no statistically significant differences in mRNA levels between the EGCG+RES and the PLA-group, although the expression pattern (direction of change) was similar for micro-array and RT-qPCR results, for all but 1 individual gene (Supplementary Table S1). As an indication of macrophage infiltration in the AT, we also measured CD68-expression by RT-qPCR. The micro-array data showed a significantly lower CD68 expression, indicative for a reduced macrophage infiltration in AT. The RT-qPCR for CD68 expression displayed a tendency in the same direction, but did not reach statistical significance.

Possible regulators of adipogenesis (β-estradiol, Prolactin), oxidative stress (Genistein, nuclear factor and erythroid 2-like 2 (NRF2)) and inflammation (TNF-α) were identified by upstream analysis of significantly altered genes (n = 763) as potentially inhibited after EGCG+RES supplementation (Supplementary Table S3). Therefore, the upstream analysis suggests that EGCG+RES supplementation leads to a decrease in adipogenesis, oxidative stress and inflammation related gene expression. In line, downstream-gene sets regulated by anti-carcinogenic and immune-suppressant drugs were identified as activated and implicate an inhibited proliferation capacity (5-Flourouracil, Trichostatin A, Gentamicin, CD437 and sirolimus).

### Adipose tissue oxidative phosphorylation (OXPHOS) protein expression

Protein expression of mitochondrial complex V tended to be decreased by EGCG+RES supplementation compared with PLA (P = 0.11, Figure 1D, Supplementary Figure S1). Complexes II, III and I+IV were not significantly affected by EGCG+RES (P = 0.31, P = 0.61, P = 0.55, respectively).

## Discussion

Numerous rodent studies have demonstrated that dietary polyphenols, including EGCG and RES, modulate AT biology [3,5]. Here, we demonstrate that supplementation of EGCG+RES for 12 weeks downregulated gene expression of pathways related to adipocyte turnover, energy metabolism, inflammation, and the immune defense. However, EGCG+RES did not induce alterations in adipocyte morphology or OXPHOS protein expression in overweight and obese subjects. Importantly, food intake and diet composition did not change throughout the intervention as assessed by 3-d food records [12].

The present study demonstrated that EGCG+RES supplementation decreased the expression of gene sets related to adipogenesis, apoptosis and lipolysis, which are major determinants of adipose tissue and lipid turnover [13–15] and a reduction in both may therefore be indicative of a reduced adipocyte turnover. This, in turn, may have caused the reduced expression of pathways related to substrate metabolism and mitochondrial function, possibly related to lower energy requirements of AT. We acknowledge that other advanced techniques such as isotope dilution may provide more direct evidence of adipose tissue turnover and should be applied in future studies [16]. Importantly, a low turnover rate of adipocytes may reflect a less flexible metabolic phenotype. Indeed, decreased adipocyte and lipid turnover has been related to hypertrophic, dysfunctional AT [13], obesity [15] and familial combined hyperlipidemia [14]. Nevertheless, the observed inactivation of pathways corresponding to adipose tissue turnover did not translate into a change in adipocyte size or AT mass in the present study (Figure 1). In line with these morphological findings, plasma metabolic profile and whole body, and AT insulin sensitivity did not change significantly after the intervention [12], despite a slight reduction in fasting insulin levels in this subgroup (Table 1).

At first glance, the present data seem in contrast to a previous study of our group, demonstrating that adipocyte size was significantly reduced, whereas fat mass was unchanged, after 30 days of RES supplementation (150 mg/d) in obese men [9]. In that study, gene expression and pathway analysis indicated that RES increased adipogenesis and enhanced lysosomal and phagosomal lipid breakdown, which may have contributed to the observed reduction in adipocyte size [9]. Importantly, however, the duration of supplementation, the lower dose of RES and the addition of EGCG as supplement in the present study might explain these opposing findings.

Intriguingly, the present data demonstrate that pathways related to oxidative stress, inflammation and the immune response showed lower expression levels in AT after EGCG+RES intake compared with PLA (Supplementary Table S2). These findings are consistent with previous studies showing anti-oxidant and anti-proliferative effects of EGCG and RES, which led to the administration of polyphenols in chemoprevention and cancer therapy [17,18]. However, due to the importance of oxidative stress and autophagy in cell and tissue homeostasis, cancer development and cardiometabolic complications [19,20], the (patho)physiological relevance of the present findings over a longer supplementation period remains to be investigated. Contrary to our observations, Konings *et al* [9]. reported an elevated gene expression for pathways related to immune response. This was interpreted as subsequent effect induced by an increased lysosomal lipid breakdown, and by the decrease in adipocyte size causing traction forces between the adipocytes and their embedding extracellular matrix. As indicated above, duration of supplementation, addition of EGCG supplementation and the inclusion of female subjects may have played a role in these differential findings. Despite the small sample size of this study, which is its major limitation, the results were consistent. If feasible, future studies would benefit from inclusion of a more thorough assessment of macrophage infiltration to characterize the inflammatory status of the AT.

We were not able to confirm the micro-array data in a statistically significant way by RT-qPCR of individual genes although the mRNA transcripts followed a similar pattern as our gene expression array data. Over the past decade micro-array technology has been established as a sensitive and robust method to detect changes in gene expression patterns [21]. Selection of a handful of genes introduces a bias in the analysis that may be avoided by using a transcriptome-wide method. In addition, the GSEA and upstream analysis does not focus on individual genes, but rather on sets of genes, i.e. pathways. Therefore, with the subtle changes (range of fold changes: −2.034 to +1.155) we observed for individual genes, potential false positive or false-negative genes have less impact on the overall results using micro-array analysis as compared to analyses of individual genes with RT-qPCR. Nevertheless, future studies are warranted to confirm these findings at transcriptional and functional level.

In conclusion, the present study illustrated that EGCG+RES supplementation for 12 weeks may induce a suppression of gene sets related to adipocyte turnover (adipogenesis and apoptosis/autophagy), inflammation and the immune system in AT in overweight and obese men and women. Although EGCG+RES did not induce any significant effects on body composition, AT morphology, lipolysis and insulin sensitivity [12], it remains to be determined how the alterations of the AT transcriptome may translate into beneficial changes in metabolic health on longer terms.

## Materials and methods

### Study design

The current study was conducted as part of a randomized, double-blind placebo-controlled intervention trial. The effects of 12 weeks EGCG+RES supplementation (282 mg/d and 80 mg/d, respectively) on insulin sensitivity and lipid metabolism were investigated in 38 overweight and obese men and premenopausal women [12]. These doses have been shown safe and effective before [9,11]. This trial was registered at clinicaltrials.gov (NCT02381145).

Before and after intervention, an abdominal subcutaneous AT biopsy was collected after an overnight fast. All subjects gave written informed consent for participation in the original study, which was approved by the Medical Ethical Committee of Maastricht University Medical Center^+^. All procedures were conducted according to the Declaration of Helsinki.

### Abdominal subcutaneous adipocyte size

AT biopsies (∼1 g) were taken before and after intervention under local anesthesia using a needle biopsy technique [22], snap-frozen using liquid nitrogen and stored at -80°C until further analyses. One portion of the biopsy was embedded in paraffin, of which sections were cut for staining (hematoxylin and eosin), digital imaging and computerized measurement of 400 individual adipocytes to determine adipocyte morphology [22].

### Adipose tissue gene and protein expression

For gene expression analysis, total RNA was extracted from frozen AT aliquots (∼300 mg) using the Trizol method (Qiagen, Venlo, Netherlands). Starting from 100 ng total RNA, fragmented and labeled ss-cDNA of each sample was hybridized onto a Human Transcriptome Array 2.0 GeneChip® (Affymetrix) [12]. Gene chip data were analyzed using the MADMAX database and analysis pipeline [23]. Probe sets were defined according to Dai *et al* [24]. Ranked gene lists created according to the intensity based moderated t-statistics [25] were used as input for Gene Set Enrichment Analysis (GSEA), which was run with 1000 permutations [26,27]. Functional data analysis was conducted on the filtered data set (interquartile range >0.2 (log2), intensity >20 on at least 5 arrays, >5 probes/gene) based upon a false discovery rate (FDR) q-value <0.05 testing for time*treatment interactions (EGCG+RES *versus* PLA). An upstream analysis was performed on the differentially expressed genes (P<0.05) in AT with Ingenuity Pathway Analysis (June 2015, QIAGEN Silicon Valley, Redwood City, CA, USA).

In addition, we determined gene expression by RT-qPCR for a selection of physiologically significant genes that were most pronounced affected (FC>1.25; p<0.05) in the micro-arrays (ATP6V1A, ATP6V1H, CD68, HSL/LIPE, LAMP2, PI4K2A, UCP2, GAPDH). Reverse transcription of 300 ng of total RNA was performed using iScript cDNA synthesis kit (Bio-Rad), and SYBR-Green based RT-qPCRs were performed using an iCycler (Bio-Rad). Reactions were performed in duplicate on the same plate in a total volume of 25 μL containing 5 μL 1:6 cDNA, 12.5 μL SYBR-Green master mix (IQ SYBR Green Supermix), and gene-specific primers (Biolegio, Nijmegen, The Netherlands; for primer sequences see Supplementary Table S4). Expression was normalized by GAPDH and the ΔΔCT method was used for calculating relative expression.

Western blot staining for mitochondrial protein complexes I&IV (NADH-dehydrogenase & cytochrome C oxidase), II (succinate-dehydrogenase), III (ubiquinol-cytochrome C reductase) and V (ATP-synthase) were performed in protein lysates from whole adipose tissue homogenates after standard SDS-PAGE (Criterion Gel System, BioRad, Veenendaal, the Netherlands) and subsequent Western blotting (Trans-Blot® SD Semi-Dry Transfer Cell, BioRad, Veenendaal, the Netherlands). OXPHOS protein are very sensitive to heating. Therefore, the samples, including the positive control, were heated only at 50°C before loaded on the gel. MitoProfile® Total OXPHOS antibody cocktail was used as primary antibody (MS601, Mitosciences, Oregon, USA), and GAPDH was used as loading contol (#2118, Rabbit-anti-Human GAPDH, Cell Signaling, Leiden, the Netherlands). Protein quantification of OXPHOS complexes was performed by Image Lab™ Software (V5.2.1. Build 11, BioRad, Veenendaal, the Netherlands) and representative blots are shown in Supplementary Figure S1.

### Statistics

Data are expressed as mean±SEM. Variables were normally distributed. Baseline differences between the EGCG+RES and PLA group were tested by Student's unpaired t-test. The effects of EGCG+RES supplementation compared with PLA were analyzed using a 2-factor (time and treatment) repeated-measures ANOVA. Gene expression data are expressed as fold-changes calculated as the change from baseline for the EGCG+RES group as compared to the change from baseline in the PLA-group: Limma (log 2 based) Fold Change = ((Mean FC: INTV_t3-INTV_t0)*(-(Mean FC: PLA_t3-PLA_t0)). Statistics was done using SPSS 19.0 (IBM Corporation, Armonk, NY, USA) for Macintosh. P<0.05 was considered as statistically significant.

## Supplementary Material

1469942_supplementary_material.zip

## Abbreviations

*AT*: Adipose tissue
*EGCG*: Epigallocatechin-gallate
*FC*: Fold change
*FDR*: False discovery rate
*GSEA*: Gene set enrichment analysis
*INTV*: Intervention
*OXPHOS*: Oxidative Phosphorylation
*PLA*: Placebo
*PCR*: Polymerase chain reaction
*RES*: Resveratrol
*RT*: Real-time

## References

[1] Lundgren M, Svensson M, Lindmark S, et al. Fat cell enlargement is an independent marker of insulin resistance and ‘hyperleptinaemia’. Diabetologia. 2007;50:625–33. doi:10.1007/s00125-006-0572-1 17216279

[2] Heinonen S, Saarinen L, Naukkarinen J, et al. Adipocyte morphology and implications for metabolic derangements in acquired obesity. Int J Obes (Lond). 2014;38:1423–31. doi:10.1038/ijo.2014.31 24549139

[3] Kim S, Jin Y, Choi Y, et al. Resveratrol exerts anti-obesity effects via mechanisms involving down-regulation of adipogenic and inflammatory processes in mice. Biochem Pharmacol. 2011;81:1343–51. doi:10.1016/j.bcp.2011.03.012 21439945

[4] Lagouge M, Argmann C, Gerhart-Hines Z, et al. Resveratrol improves mitochondrial function and protects against metabolic disease by activating SIRT1 and PGC-1alpha. Cell. 2006;127:1109–22. Epub 2006/11/23. doi:10.1016/j.cell.2006.11.013 17112576

[5] Lee MS, Kim CT, Kim Y Green tea (-)-epigallocatechin-3-gallate reduces body weight with regulation of multiple genes expression in adipose tissue of diet-induced obese mice. Ann Nutr Metab. 2009;54:151–7. doi:10.1159/000214834 19390166

[6] Wolfram S, Raederstorff D, Wang Y, et al. TEAVIGO (epigallocatechin gallate) supplementation prevents obesity in rodents by reducing adipose tissue mass. Ann Nutr Metab. 2005;49:54–63. doi:10.1159/000084178 15735368

[7] Yoshino J, Conte C, Fontana L, et al. Resveratrol supplementation does not improve metabolic function in nonobese women with normal glucose tolerance. Cell Metab. 2012;16:658–64. doi:10.1016/j.cmet.2012.09.015 23102619 PMC3496026

[8] Mielgo-Ayuso J, Barrenechea L, Alcorta P, et al. Effects of dietary supplementation with epigallocatechin-3-gallate on weight loss, energy homeostasis, cardiometabolic risk factors and liver function in obese women: randomised, double-blind, placebo-controlled clinical trial. Br J Nutr. 2014;111:1263–71. doi:10.1017/S0007114513003784 24299662

[9] Konings E, Timmers S, Boekschoten MV, et al. The effects of 30 days resveratrol supplementation on adipose tissue morphology and gene expression patterns in obese men. Int J Obes (Lond). 2014;38:470–3. doi:10.1038/ijo.2013.155 23958793

[10] Most J, van Can JG, van Dijk JW, et al. A 3-day EGCG-supplementation reduces interstitial lactate concentration in skeletal muscle of overweight subjects. Sci Rep. 2015;5:17896. doi:10.1038/srep17896 26647963 PMC4673403

[11] Most J, Goossens GH, Jocken JW, et al. Short-term supplementation with a specific combination of dietary polyphenols increases energy expenditure and alters substrate metabolism in overweight subjects. Int J Obes (Lond). 2014;38:698–706. doi:10.1038/ijo.2013.231 24317366

[12] Most J, Timmers S, Warnke I, et al. Combined epigallocatechin-3-gallate and resveratrol supplementation for 12 wk increases mitochondrial capacity and fat oxidation, but not insulin sensitivity, in obese humans: a randomized controlled trial. Am J Clin Nutr. 2016;104:215–27. Epub 2016/05/20. doi:10.3945/ajcn.115.122937 27194304

[13] Arner E, Westermark PO, Spalding KL, et al. Adipocyte turnover: relevance to human adipose tissue morphology. Diabetes. 2010;59:105–9. doi:10.2337/db09-0942 19846802 PMC2797910

[14] Arner P, Bernard S, Salehpour M, et al. Dynamics of human adipose lipid turnover in health and metabolic disease. Nature. 2011;478:110–3. doi:10.1038/nature10426 21947005 PMC3773935

[15] Ryden M, Andersson DP, Bernard S, et al. Adipocyte triglyceride turnover and lipolysis in lean and overweight subjects. J Lipid Res. 2013;54:2909–13. doi:10.1194/jlr.M040345 23899442 PMC3770103

[16] White UA, Tchoukalova YD Implications of 2H-labeling of DNA protocol to measure in vivo cell turnover in adipose tissue. Adipocyte. 2012;1:242–5. doi:10.4161/adip.20817 23700539 PMC3609104

[17] Goswami SK, Das DK Resveratrol and chemoprevention. Cancer Lett. 2009;284:1–6. doi:10.1016/j.canlet.2009.01.041 19261378

[18] Yang CS, Wang X, Lu G, et al. Cancer prevention by tea: animal studies, molecular mechanisms and human relevance. Nat Rev Cancer. 2009;9:429–39. doi:10.1038/nrc2641 19472429 PMC2829848

[19] Kim KH, Lee MS Autophagy–a key player in cellular and body metabolism. Nat Rev Endocrinol. 2014;10:322–37. doi:10.1038/nrendo.2014.35 24663220

[20] Gorrini C, Harris IS, Mak TW Modulation of oxidative stress as an anticancer strategy. Nat Rev Drug Discov. 2013;12:931–47. doi:10.1038/nrd4002 24287781

[21] Morey JS, Ryan JC, Van Dolah FM Microarray validation: factors influencing correlation between oligonucleotide microarrays and real-time PCR. Biol Proced Online. 2006;8:175–93. doi:10.1251/bpo126 17242735 PMC1779618

[22] Goossens GH, Bizzarri A, Venteclef N, et al. Increased adipose tissue oxygen tension in obese compared with lean men is accompanied by insulin resistance, impaired adipose tissue capillarization, and inflammation. Circulation. 2011;124:67–76. doi:10.1161/CIRCULATIONAHA.111.027813 21670228

[23] Lin K, Kools H, de Groot PJ, et al. MADMAX - Management and analysis database for multiple ∼omics experiments. J Integr Bioinform. 2011;8:160. doi:10.1515/jib-2011-160 21778530

[24] Dai MH, Wang PL, Boyd AD, et al. Evolving gene/transcript definitions significantly alter the interpretation of GeneChip data. Nucleic Acids Research. 2005;33:e175. doi:10.1093/nar/gni179 PMC1283542 16284200

[25] Sartor MA, Tomlinson CR, Wesselkamper SC, et al. Intensity-based hierarchical Bayes method improves testing for differentially expressed genes in microarray experiments. Bmc Bioinformatics. 2006;7:538. doi:10.1186/1471-2105-7-538 PMC1781470 17177995

[26] Subramanian A, Tamayo P, Mootha VK, et al. Gene set enrichment analysis: A knowledge-based approach for interpreting genome-wide expression profiles. Proceedings of the National Academy of Sciences of the United States of America. 2005;102:15545–50. doi:10.1073/pnas.0506580102 16199517 PMC1239896

[27] Mootha VK, Lindgren CM, Eriksson KF, et al. PGC-1 alpha-responsive genes involved in oxidative phosphorylation are coordinately downregulated in human diabetes. Nature Genetics. 2003;34:267–73. doi:10.1038/ng1180 12808457

