# Supplementary material for: The effects of polyphenol supplementation on adipose tissue morphology and gene expression in overweight and obese humans
Source: Adipocyte. 2018 May 22;7(3):190–6. doi: 10.1080/21623945.2018.1469942 (PMC6224187; doi:10.1080/21623945.2018.1469942)
Supplement: 1469942_supplementary_material.zip [file kadi-07-03-1469942-s001.zip › 1469942_supplementary material/Dietary polyphenols & adipose tissue_Supplementary Figure.docx]

# The effects of polyphenol supplementation on adipose tissue morphology and gene expression in overweight and obese humans

Jasper Most^1,4^, Ines Warnke^2,4^, Mark Boekschoten^3^, Johan W.E. Jocken^1^, Philip de Groot^3^, Angelika Friedel^2^, Igor Bendik^2^, Gijs H. Goossens^1^, Ellen E. Blaak^1^

## Supplementary Figures

| **A**  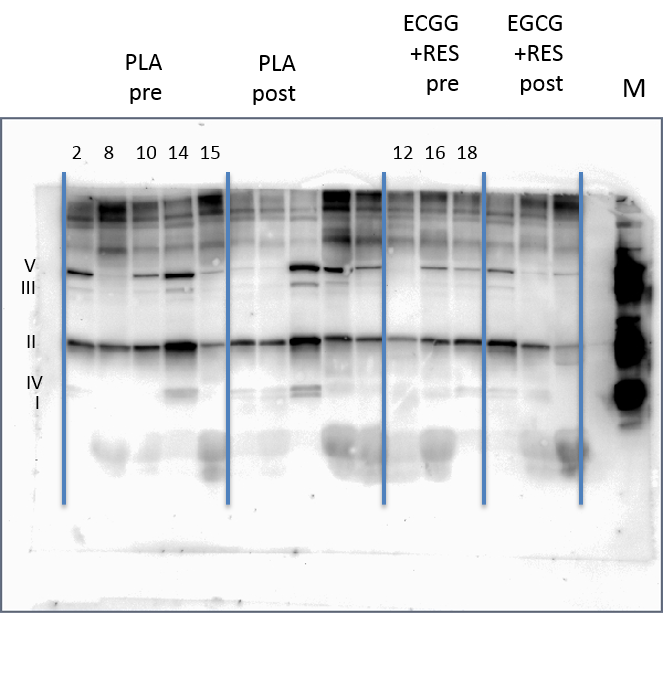 |
| --- |
| **B**  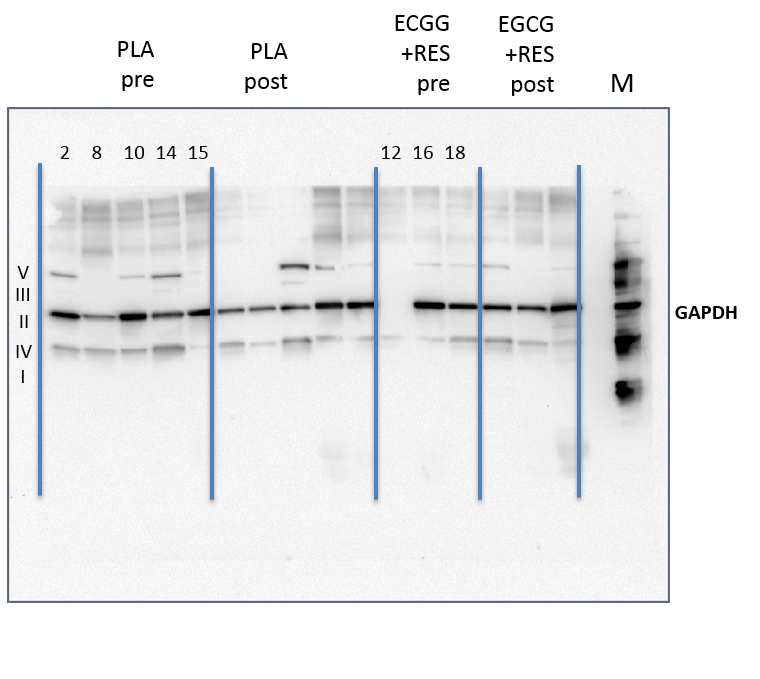 |

### Supplementary Figure S1: OXPHOS protein expression in abdominal subcutaneous adipose tissue before (pre) and after (post) intervention in PLA subjects (2,8, 10, 14 and 15) or EGCG+RES subjects (12, 16 and 18). A) Complexes I&IV (NADH-dehydrogenase & cytochrome C oxidase), II (succinate-dehydrogenase), III (ubiquinol-cytochrome C reductase), V (ATP-synthase), M (SDS-PAGE Molecular Weight Standard Broad Range) showed predicted bands between 50 and 20 kDa. B) GAPDH was used as loading control and showed a band at 37kDa. Shown are images of two representative western blots (in total: EGCG+RES, n=5; PLA n=9).
