# Supplementary material for: The effects of polyphenol supplementation on adipose tissue morphology and gene expression in overweight and obese humans
Source: Adipocyte. 2018 May 22;7(3):190–6. doi: 10.1080/21623945.2018.1469942 (PMC6224187; doi:10.1080/21623945.2018.1469942)
Supplement: 1469942_supplementary_material.zip [file kadi-07-03-1469942-s001.zip › 1469942_supplementary material/Dietary polyphenols & adipose tissue_Supplementary Tables.docx]

Supplementary Table S1. Selection of the 100 most up- and downregulated genes

|  | Gene | Entrez ID | Description | Fold-change (MA) | P | Fold-change (RT-qPCR) | P |
| --- | --- | --- | --- | --- | --- | --- | --- |
| Most down-regulated, sorted by FC | | | | | | | |
|  | MS4A6E | 245802 | membrane-spanning 4-domains, subfamily A, member 6E | -2.042 | 0.031 |  |  |
|  | GPR183 | 1880 | G protein-coupled receptor 183 | -1.62 | 0.006 |  |  |
|  | SPESP1 | 246777 | sperm equatorial segment protein 1 | -1.494 | 0.004 |  |  |
|  | RNASE6 | 6039 | ribonuclease, RNase A family, k6 | -1.489 | 0.026 |  |  |
|  | LOC101927405 | 101927405 | uncharacterized LOC101927405 | -1.482 | 0.02 |  |  |
|  | CD68 | 968 | CD68 molecule | -1.455 | 0.041 | -1.033 | 0.779 |
|  | LOC102723793 | 102723793 | uncharacterized LOC102723793 | -1.429 | 0.023 |  |  |
|  | LOC102724528 | 102724528 | uncharacterized LOC102724528 | -1.42 | 0.02 |  |  |
|  | GPNMB | 10457 | glycoprotein (transmembrane) nmb | -1.412 | 0.05 |  |  |
|  | SLMO2 | 51012 | slowmo homolog 2 (Drosophila) | -1.411 | 0.046 |  |  |
|  | CXCL16 | 58191 | chemokine (C-X-C motif) ligand 16 | -1.409 | 0.023 |  |  |
|  | UCP2 | 7351 | uncoupling protein 2 (mitochondrial, proton carrier) | -1.4 | 0.004 | -1.347 | 0.655 |
|  | CTSD | 1509 | cathepsin D | -1.399 | 0.025 |  |  |
|  | PRDX3 | 10935 | peroxiredoxin 3 | -1.395 | 0.01 |  |  |
|  | LOC101929216 | 101929216 | uncharacterized LOC101929216 | -1.394 | 0.047 |  |  |
|  | SLC31A1 | 1317 | solute carrier family 31 (copper transporter), member 1 | -1.393 | 0.005 |  |  |
|  | GPRIN3 | 285513 | GPRIN family member 3 | -1.388 | 0.003 |  |  |
|  | TMSB4X | 7114 | thymosin beta 4, X-linked | -1.375 | 0.004 |  |  |
|  | SAMHD1 | 25939 | SAM domain and HD domain 1 | -1.375 | 0.04 |  |  |
|  | MS4A7 | 58475 | membrane-spanning 4-domains, subfamily A, member 7 | -1.373 | 0.029 |  |  |
|  | S100A4 | 6275 | S100 calcium binding protein A4 | -1.368 | 0.019 |  |  |
|  | MMP19 | 4327 | matrix metallopeptidase 19 | -1.366 | 0.039 |  |  |
|  | MIR4435-1HG | 541471 | MIR4435-1 host gene (non-protein coding) | -1.364 | 0.018 |  |  |
|  | RPL23A | 6147 | ribosomal protein L23a | -1.363 | 0.028 |  |  |
|  | IFI6 | 2537 | interferon, alpha-inducible protein 6 | -1.36 | 0.039 |  |  |
|  | CTSB | 1508 | cathepsin B | -1.354 | 0.043 |  |  |
|  | HAVCR2 | 84868 | hepatitis A virus cellular receptor 2 | -1.354 | 0.01 |  |  |
|  | TOMM6 | 100188893 | translocase of outer mitochondrial membrane 6 homolog (yeast) | -1.347 | 0.006 |  |  |
|  | SOAT1 | 6646 | sterol O-acyltransferase 1 | -1.346 | 0.015 |  |  |
|  | GM2A | 2760 | GM2 ganglioside activator | -1.345 | 0.008 |  |  |
|  | GLB1 | 2720 | galactosidase, beta 1 | -1.345 | 0.007 |  |  |
|  | MS4A4A | 51338 | membrane-spanning 4-domains, subfamily A, member 4A | -1.342 | 0.032 |  |  |
|  | ANXA1 | 301 | annexin A1 | -1.342 | 0.023 |  |  |
|  | MOSPD1 | 56180 | motile sperm domain containing 1 | -1.34 | 0.002 |  |  |
|  | CDC42SE2 | 56990 | CDC42 small effector 2 | -1.338 | 0.023 |  |  |
|  | SGTB | 54557 | small glutamine-rich tetratricopeptide repeat (TPR)-containing, beta | -1.336 | 0.003 |  |  |
|  | ATP6V0E1 | 8992 | ATPase, H+ transporting, lysosomal 9kDa, V0 subunit e1 | -1.335 | 0.004 |  |  |
|  | MFSD1 | 64747 | major facilitator superfamily domain containing 1 | -1.334 | 0.003 |  |  |
|  | DDT | 1652 | D-dopachrome tautomerase | -1.332 | 0.01 |  |  |
|  | PLP2 | 5355 | proteolipid protein 2 (colonic epithelium-enriched) | -1.332 | 0.05 |  |  |
|  | GLIPR2 | 152007 | GLI pathogenesis-related 2 | -1.33 | 0.022 |  |  |
|  | PPT1 | 5538 | palmitoyl-protein thioesterase 1 | -1.329 | 0.027 |  |  |
|  | SRD5A3 | 79644 | steroid 5 alpha-reductase 3 | -1.321 | 0.007 |  |  |
|  | SNX2 | 6643 | sorting nexin 2 | -1.321 | 0.011 |  |  |
|  | ERO1L | 30001 | ERO1-like (S. cerevisiae) | -1.321 | 0.022 |  |  |
|  | RPS15A | 6210 | ribosomal protein S15a | -1.318 | 0.033 |  |  |
|  | ID2 | 3398 | inhibitor of DNA binding 2, dominant negative helix-loop-helix protein | -1.314 | 0.01 |  |  |
|  | GLA | 2717 | galactosidase, alpha | -1.313 | 0.002 |  |  |
|  | MME | 4311 | membrane metallo-endopeptidase | -1.313 | 0.039 |  |  |
|  | LRRC37B | 114659 | leucine rich repeat containing 37B | -1.308 | 0.035 |  |  |
|  | HIST1H4E | 8367 | histone cluster 1, H4e | -1.308 | 0.017 |  |  |
|  | SLC31A2 | 1318 | solute carrier family 31 (copper transporter), member 2 | -1.307 | 0.008 |  |  |
|  | SH3BGRL | 6451 | SH3 domain binding glutamate-rich protein like | -1.305 | 0.006 |  |  |
|  | TMED7 | 51014 | transmembrane emp24 protein transport domain containing 7 | -1.305 | 0.012 |  |  |
|  | CAPZA1 | 829 | capping protein (actin filament) muscle Z-line, alpha 1 | -1.304 | 0.007 |  |  |
|  | OSBPL11 | 114885 | oxysterol binding protein-like 11 | -1.303 | 0.019 |  |  |
|  | NDUFA4 | 4697 | NDUFA4, mitochondrial complex associated | -1.303 | 0.03 |  |  |
|  | RAB7A | 7879 | RAB7A, member RAS oncogene family | -1.302 | 0.012 |  |  |
|  | ATP5C1 | 509 | ATP synthase, H+ transporting, mitochondrial F1 complex, gamma polypeptide 1 | -1.301 | 0.041 |  |  |
|  | MOB1A | 55233 | MOB kinase activator 1A | -1.299 | 0.004 |  |  |
|  | GRN | 2896 | granulin | -1.298 | 0.021 |  |  |
|  | CPA3 | 1359 | carboxypeptidase A3 (mast cell) | -1.297 | 0.048 |  |  |
|  | HSD17B12 | 51144 | hydroxysteroid (17-beta) dehydrogenase 12 | -1.296 | 0.044 |  |  |
|  | PGD | 5226 | phosphogluconate dehydrogenase | -1.296 | 0.012 |  |  |
|  | DKFZP586I1420 | 222161 | uncharacterized protein DKFZp586I1420 | -1.296 | 0.03 |  |  |
|  | ANKRD49 | 54851 | ankyrin repeat domain 49 | -1.295 | 0.043 |  |  |
|  | SUMO4 | 387082 | small ubiquitin-like modifier 4 | -1.293 | 0.007 |  |  |
|  | ZBTB6 | 10773 | zinc finger and BTB domain containing 6 | -1.292 | 0.004 |  |  |
|  | ATP5J2 | 9551 | ATP synthase, H+ transporting, mitochondrial Fo complex, subunit F2 | -1.29 | 0.011 |  |  |
|  | LPCAT2 | 54947 | lysophosphatidylcholine acyltransferase 2 | -1.29 | 0.007 |  |  |
|  | MBTPS2 | 51360 | membrane-bound transcription factor peptidase, site 2 | -1.286 | 0.022 |  |  |
|  | TMEM88 | 92162 | transmembrane protein 88 | -1.286 | 0.02 |  |  |
|  | SDHB | 6390 | succinate dehydrogenase complex, subunit B, iron sulfur (Ip) | -1.284 | 0.01 |  |  |
|  | CCDC88A | 55704 | coiled-coil domain containing 88A | -1.284 | 0.046 |  |  |
|  | TPP1 | 1200 | tripeptidyl peptidase I | -1.284 | 0.016 |  |  |
|  | PI4K2A | 55361 | phosphatidylinositol 4-kinase type 2 alpha | -1.283 | 0.001 | -1.377 | 0.6 |
|  | ECH1 | 1891 | enoyl CoA hydratase 1, peroxisomal | -1.282 | 0.036 |  |  |
|  | BLVRB | 645 | biliverdin reductase B (flavin reductase (NADPH)) | -1.281 | 0.047 |  |  |
|  | LAMP2 | 3920 | lysosomal-associated membrane protein 2 | -1.279 | 0.008 | -2.034 | 0.111 |
|  | LACTB | 114294 | lactamase, beta | -1.276 | 0.01 |  |  |
|  | MBNL1 | 4154 | muscleblind-like splicing regulator 1 | -1.275 | 0.003 |  |  |
|  | SERPINB8 | 5271 | serpin peptidase inhibitor, clade B (ovalbumin), member 8 | -1.275 | 0.004 |  |  |
|  | LIPE | 3991 | lipase, hormone-sensitive | -1.271 | 0.019 | 1.155 | 0.576 |
|  | GNS | 2799 | glucosamine (N-acetyl)-6-sulfatase | -1.271 | 0.014 |  |  |
|  | POLR2B | 5431 | polymerase (RNA) II (DNA directed) polypeptide B, 140kDa | -1.27 | 0.031 |  |  |
|  | PSEN1 | 5663 | presenilin 1 | -1.269 | 0.002 |  |  |
|  | CDC26 | 246184 | cell division cycle 26 | -1.268 | 0.048 |  |  |
|  | ARL6IP5 | 10550 | ADP-ribosylation factor-like 6 interacting protein 5 | -1.268 | 0.01 |  |  |
|  | RPS25 | 6230 | ribosomal protein S25 | -1.267 | 0.011 |  |  |
|  | CSTF2T | 23283 | cleavage stimulation factor, 3' pre-RNA, subunit 2, 64kDa, tau variant | -1.267 | 0.02 |  |  |
|  | HPS3 | 84343 | Hermansky-Pudlak syndrome 3 | -1.267 | 0.008 |  |  |
|  | C4B | 721 | complement component 4B (Chido blood group) | -1.266 | 0.021 |  |  |
|  | TMEM258 | 746 | transmembrane protein 258 | -1.266 | 0.047 |  |  |
|  | FTL | 2512 | ferritin, light polypeptide | -1.264 | 0.018 |  |  |
|  | HLA-DRA | 3122 | major histocompatibility complex, class II, DR alpha | -1.264 | 0.049 |  |  |
|  | PPP1R15B | 84919 | protein phosphatase 1, regulatory subunit 15B | -1.264 | 0.01 |  |  |
|  | ZNF267 | 10308 | zinc finger protein 267 | -1.263 | 0.041 |  |  |
|  | NCSTN | 23385 | nicastrin | -1.261 | 0.007 |  |  |
|  | SUPT7L | 9913 | suppressor of Ty 7 (S. cerevisiae)-like | -1.258 | 0.009 |  |  |
|  | RAB8A | 4218 | RAB8A, member RAS oncogene family | -1.257 | 0.026 |  |  |
|  | ATP6V1A | 523 | ATPase, H+ transporting, lysosomal 70kDa, V1 subunit A | -1.255 | 0.009 | -1.079 | 0.56 |
|  | ATP6V1H | 51606 | ATPase, H+ transporting, lysosomal 50/57kDa, V1 subunit H | -1.25 | 0.004 | -1.074 | 0.982 |
|  |  |  |  |  |  |  |  |
| Most up-regulated, sorted by FC | | | | | | | |
|  | RNU6-23P | 100873755 | RNA, U6 small nuclear 23, pseudogene | 2.057 | 0.013 |  |  |
|  | MIR6875 | 102466755 | microRNA 6875 | 1.79 | 0.002 |  |  |
|  | MIR4442 | 100616477 | microRNA 4442 | 1.688 | 0.004 |  |  |
|  | MIR3689D2 | 100616344 | microRNA 3689d-2 | 1.623 | 0.03 |  |  |
|  | TRM-CAT1-1 | 100189201 | transfer RNA-Met (CAT) 1-1 | 1.611 | 0.011 |  |  |
|  | MIR6873 | 102466754 | microRNA 6873 | 1.59 | 0.004 |  |  |
|  | TRS-AGA3-1 | 790951 | transfer RNA-Ser (AGA) 3-1 | 1.583 | 0.011 |  |  |
|  | SNORA71E | 677821 | small nucleolar RNA, H/ACA box 71E | 1.571 | 0.01 |  |  |
|  | RNASEK-C17orf49 | 100529209 | RNASEK-C17orf49 readthrough | 1.565 | 0.003 |  |  |
|  | MIR936 | 100126326 | microRNA 936 | 1.547 | 0.001 |  |  |
|  | TRM-CAT6-1 | 100189226 | transfer RNA-Met (CAT) 6-1 | 1.52 | 0.025 |  |  |
|  | ERCC6-PGBD3 | 101243544 | ERCC6-PGBD3 readthrough | 1.513 | 0.006 |  |  |
|  | MIR133B | 442890 | microRNA 133b | 1.505 | 0.002 |  |  |
|  | TRK-CTT6-1 | 100188995 | transfer RNA-Lys (CTT) 6-1 | 1.5 | 0.044 |  |  |
|  | CT47B1 | 643311 | cancer/testis antigen family 47, member B1 | 1.498 | 0.001 |  |  |
|  | LOC728554 | 728554 | THO complex 3 pseudogene | 1.485 | 0.007 |  |  |
|  | LOC101929974 | 101929974 | uncharacterized LOC101929974 | 1.481 | 0.008 |  |  |
|  | TRS-CGA4-1 | 100189152 | transfer RNA-Ser (CGA) 4-1 | 1.476 | 0.014 |  |  |
|  | IGLJ4 | 28830 | immunoglobulin lambda joining 4 (non-functional) | 1.474 | 0.015 |  |  |
|  | MIR3147 | 100422939 | microRNA 3147 | 1.473 | 0.01 |  |  |
|  | LOC729468 | 729468 | putative PGM5-like protein 1 | 1.463 | 0.007 |  |  |
|  | TRAJ7 | 28748 | T cell receptor alpha joining 7 | 1.455 | 0.007 |  |  |
|  | MIR1292 | 100302138 | microRNA 1292 | 1.448 | 0.033 |  |  |
|  | MIR1185-2 | 100302209 | microRNA 1185-2 | 1.438 | 0.01 |  |  |
|  | TRG-CCC5-1 | 100189045 | transfer RNA-Gly (CCC) 5-1 | 1.431 | 0.012 |  |  |
|  | LOC101927708 | 101927708 | uncharacterized LOC101927708 | 1.43 | 0.024 |  |  |
|  | EIF4EBP3 | 8637 | eukaryotic translation initiation factor 4E binding protein 3 | 1.424 | 0.021 |  |  |
|  | LINC00273 | 649159 | long intergenic non-protein coding RNA 273 | 1.423 | 0.015 |  |  |
|  | ARL2-SNX15 | 100528018 | ARL2-SNX15 readthrough (NMD candidate) | 1.42 | 0.015 |  |  |
|  | TRR-TCT5-1 | 100189012 | transfer RNA-Arg (TCT) 5-1 | 1.413 | 0.013 |  |  |
|  | DNAJB5-AS1 | 101926900 | DNAJB5 antisense RNA 1 (head to head) | 1.411 | 0.003 |  |  |
|  | IGLV3-19 | 28797 | immunoglobulin lambda variable 3-19 | 1.41 | 0.014 |  |  |
|  | TRA-CGC4-1 | 100189033 | transfer RNA-Ala (CGC) 4-1 | 1.408 | 0.043 |  |  |
|  | TMEM256-PLSCR3 | 100529211 | TMEM256-PLSCR3 readthrough (NMD candidate) | 1.404 | 0.005 |  |  |
|  | TRD-GTC3-1 | 100189200 | transfer RNA-Asp (GTC) 3-1 | 1.397 | 0.042 |  |  |
|  | IGLV4-69 | 28784 | immunoglobulin lambda variable 4-69 | 1.397 | 0.011 |  |  |
|  | LOC100652807 | 100652807 | putative uncharacterized protein C3orf53-like | 1.396 | 0.012 |  |  |
|  | MIR27A | 407018 | microRNA 27a | 1.391 | 0.006 |  |  |
|  | LOC101928768 | 101928768 | uncharacterized LOC101928768 | 1.387 | 0.021 |  |  |
|  | GOLGA8T | 653075 | golgin A8 family, member T | 1.378 | 0.011 |  |  |
|  | MIR4649 | 100616346 | microRNA 4649 | 1.378 | 0.03 |  |  |
|  | FAM25A | 643161 | family with sequence similarity 25, member A | 1.376 | 0.005 |  |  |
|  | MIR23B | 407011 | microRNA 23b | 1.375 | 0.015 |  |  |
|  | LOC101929280 | 101929280 | uncharacterized LOC101929280 | 1.373 | 0.005 |  |  |
|  | SMG1P3 | 100271836 | SMG1 pseudogene 3 | 1.372 | 0.002 |  |  |
|  | LOC113230 | 113230 | uncharacterized protein LOC113230 | 1.372 | 0 |  |  |
|  | MIR6734 | 102466723 | microRNA 6734 | 1.37 | 0.026 |  |  |
|  | TRW-CCA5-1 | 100189190 | transfer RNA-Trp (CCA) 5-1 | 1.37 | 0.004 |  |  |
|  | TRBJ2-6 | 28623 | T cell receptor beta joining 2-6 | 1.367 | 0.043 |  |  |
|  | MIR769 | 768217 | microRNA 769 | 1.364 | 0.04 |  |  |
|  | MIR3155A | 100422989 | microRNA 3155a | 1.363 | 0.007 |  |  |
|  | RPP21 | 79897 | ribonuclease P/MRP 21kDa subunit | 1.354 | 0.027 |  |  |
|  | LOC101927260 | 101927260 | uncharacterized LOC101927260 | 1.352 | 0.001 |  |  |
|  | HECW2 | 57520 | HECT, C2 and WW domain containing E3 ubiquitin protein ligase 2 | 1.352 | 0.041 |  |  |
|  | MIR4532 | 100616353 | microRNA 4532 | 1.35 | 0.004 |  |  |
|  | OTUD6A | 139562 | OTU deubiquitinase 6A | 1.35 | 0.006 |  |  |
|  | TRV-AAC2-1 | 100188992 | transfer RNA-Val (AAC) 2-1 | 1.35 | 0.025 |  |  |
|  | LOC102288414 | 102288414 | uncharacterized LOC102288414 | 1.348 | 0.007 |  |  |
|  | ZNF321P | 399669 | zinc finger protein 321, pseudogene | 1.345 | 0 |  |  |
|  | TRAJ12 | 28743 | T cell receptor alpha joining 12 | 1.344 | 0.048 |  |  |
|  | MYH11 | 4629 | myosin, heavy chain 11, smooth muscle | 1.343 | 0.024 |  |  |
|  | LOC101929536 | 101929536 | atherin-like | 1.343 | 0.015 |  |  |
|  | MIR4479 | 100616480 | microRNA 4479 | 1.339 | 0.027 |  |  |
|  | LOC101927441 | 101927441 | uncharacterized LOC101927441 | 1.338 | 0.027 |  |  |
|  | MIR3605 | 100500853 | microRNA 3605 | 1.336 | 0.013 |  |  |
|  | PTCD1 | 26024 | pentatricopeptide repeat domain 1 | 1.334 | 0.008 |  |  |
|  | LINC00235 | 64493 | long intergenic non-protein coding RNA 235 | 1.334 | 0.016 |  |  |
|  | MIR3162 | 100422880 | microRNA 3162 | 1.333 | 0.002 |  |  |
|  | MIR718 | 100313781 | microRNA 718 | 1.332 | 0.007 |  |  |
|  | MIR1469 | 100302258 | microRNA 1469 | 1.331 | 0.003 |  |  |
|  | MIR933 | 100126350 | microRNA 933 | 1.33 | 0.006 |  |  |
|  | KRTAP19-7 | 337974 | keratin associated protein 19-7 | 1.33 | 0.033 |  |  |
|  | MIR551B | 693136 | microRNA 551b | 1.329 | 0.03 |  |  |
|  | EDN1 | 1906 | endothelin 1 | 1.329 | 0.046 |  |  |
|  | SMG1P1 | 641298 | SMG1 pseudogene 1 | 1.328 | 0.025 |  |  |
|  | KRTAP19-5 | 337972 | keratin associated protein 19-5 | 1.327 | 0 |  |  |
|  | LOC102724965 | 102724965 | collagen alpha-2(I) chain-like | 1.327 | 0.006 |  |  |
|  | LOC101928233 | 101928233 | uncharacterized LOC101928233 | 1.326 | 0.002 |  |  |
|  | KRTAP21-1 | 337977 | keratin associated protein 21-1 | 1.322 | 0.04 |  |  |
|  | LOC101928038 | 101928038 | CDK5 and ABL1 enzyme substrate 2-like | 1.322 | 0.006 |  |  |
|  | MIR657 | 724027 | microRNA 657 | 1.322 | 0.039 |  |  |
|  | MIR125A | 406910 | microRNA 125a | 1.321 | 0.005 |  |  |
|  | SDCBP2 | 27111 | syndecan binding protein (syntenin) 2 | 1.32 | 0.005 |  |  |
|  | RPRM | 56475 | reprimo, TP53 dependent G2 arrest mediator candidate | 1.319 | 0.028 |  |  |
|  | TRG-GCC5-1 | 100189220 | transfer RNA-Gly (GCC) 5-1 | 1.319 | 0.003 |  |  |
|  | LOC101928941 | 101928941 | uncharacterized LOC101928941 | 1.318 | 0.023 |  |  |
|  | LOC102723772 | 102723772 | uncharacterized LOC102723772 | 1.313 | 0.001 |  |  |
|  | IGLV3-22 | 28795 | immunoglobulin lambda variable 3-22 (gene/pseudogene) | 1.313 | 0.009 |  |  |
|  | MIR4531 | 100616355 | microRNA 4531 | 1.311 | 0.009 |  |  |
|  | VENTXP7 | 391518 | VENT homeobox pseudogene 7 | 1.31 | 0.021 |  |  |
|  | MIR4673 | 100616242 | microRNA 4673 | 1.309 | 0.048 |  |  |
|  | GOLGA2P10 | 80154 | golgin A2 pseudogene 10 | 1.308 | 0.024 |  |  |
|  | MIR1470 | 100302127 | microRNA 1470 | 1.306 | 0.018 |  |  |
|  | MIR921 | 100126349 | microRNA 921 | 1.305 | 0.029 |  |  |
|  | TNFRSF6B | 8771 | tumor necrosis factor receptor superfamily, member 6b, decoy | 1.305 | 0.004 |  |  |
|  | TRR-TCT4-1 | 100189166 | transfer RNA-Arg (TCT) 4-1 | 1.304 | 0.012 |  |  |
|  | NKX6-2 | 84504 | NK6 homeobox 2 | 1.303 | 0.03 |  |  |
|  | GTF2A1L | 11036 | general transcription factor IIA, 1-like | 1.302 | 0.021 |  |  |
|  | LOC101929384 | 101929384 | uncharacterized LOC101929384 | 1.301 | 0.021 |  |  |
|  | MIR764 | 100313838 | microRNA 764 | 1.297 | 0.005 |  |  |

List of the most up- and downregulated genes as analyzed by micro-array (both, n=100) and PCR (n=5) and of 5 additional genes based on their physiological relevance to our hypothesis. Limma fold-changes are calculated as fold-change from baseline for the EGCG+RES group as compared to the fold-change from baseline in the PLA-group, and P-values are raw p-values from regularised paired t-test (FC(EGCG+RES)*FC(PLA)).

Supplementary Table S2. Complete list of up- and downregulated pathways

|  | Pathway | NES | q | Source |
| --- | --- | --- | --- | --- |
| Cell cycle | | | | |
|  | APC.C.MEDIATED.DEGRADATION.OF.CELL.CYCLE.PROTEINS | -2.85 | <0.001 | WP1782 |
|  | BDNF.SIGNALING.PATHWAY | -1.75 | 0.022 | WP2380 |
|  | CELL.CYCLE | -2.29 | <0.001 | WP179 |
|  | CELL.CYCLE | -2.36 | <0.001 | KEGG |
|  | CELLCYCLE | -1.87 | 0.010 | BIOC |
|  | CELL.CYCLE.CHECKPOINTS | -3.02 | <0.001 | WP1775 |
|  | AKAPCENTROSOME | -1.71 | 0.029 | BIOC |
|  | EGF.EGFR.SIGNALING.PATHWAY | -2.31 | <0.001 | WP437 |
|  | EGF | -1.72 | 0.028 | BIOC |
|  | EUKARYOTIC.TRANSCRIPTION.INITIATION | -2.48 | <0.001 | WP405 |
|  | EUKARYOTIC.TRANSLATION.ELONGATION | -1.92 | 0.007 | WP1811 |
|  | EUKARYOTIC.TRANSLATION.INITIATION | -2.10 | 0.001 | WP1812 |
|  | EUKARYOTIC.TRANSLATION.TERMINATION | -1.81 | 0.015 | WP1813 |
|  | G1.TO.S.CELL.CYCLE.CONTROL | -1.90 | 0.008 | WP45 |
|  | P53 | -1.65 | 0.039 | BIOC |
|  | G13.SIGNALING.PATHWAY | -2.32 | <0.001 | WP524 |
|  | G1 | -1.98 | 0.004 | BIOC |
|  | M.G1.TRANSITION | -2.80 | <0.001 | WP2785 |
|  | RACCYCD | -1.72 | 0.026 | BIOC |
|  | P27 | -1.70 | 0.029 | BIOC |
|  | MEIOTIC.RECOMBINATION | -1.75 | 0.023 | WP2698 |
|  | MITOTIC.G1.G1.S.PHASES | -2.67 | <0.001 | WP1858 |
|  | MITOTIC.G2.G2.M.PHASES | -2.30 | <0.001 | WP1859 |
|  | MITOTIC.METAPHASE.AND.ANAPHASE | -2.85 | <0.001 | WP2757 |
|  | MITOTIC.PROMETAPHASE | -2.01 | 0.003 | WP2652 |
|  | MITOTIC.PROPHASE | -2.50 | <0.001 | WP2654 |
|  | MITOTIC.TELOPHASE.CYTOKINESIS | -1.68 | 0.033 | WP2765 |
|  | PHASE.II.CONJUGATION | -1.93 | 0.006 | WP1880 |
|  | RAF.MAP.KINASE.CASCADE | -1.84 | 0.012 | WP2735 |
|  | SPRY | -1.67 | 0.036 | BIOC |
|  | S.PHASE | -2.74 | <0.001 | WP2772 |
| Cellular maintenance | | | | |
|  | ANDROGEN.RECEPTOR.SIGNALING.PATHWAY | -2.51 | <0.001 | WP138 |
|  | CIRCADIAN.CLOCK | -1.81 | 0.015 | WP1797 |
|  | CIRCADIAN.RHYTHM | -2.29 | <0.001 | KEGG |
|  | DIURNALLY.REGULATED.GENES.WITH.CIRCADIAN.ORTHOLOGS | -2.25 | <0.001 | WP410 |
|  | FCGAMMA.RECEPTOR.FCGR.DEPENDENT.PHAGOCYTOSIS. | -2.23 | <0.001 | WP2719 |
|  | FC.GAMMA.R.MEDIATED.PHAGOCYTOSIS | -1.96 | 0.005 | KEGG |
|  | PHAGOSOME | -2.79 | <0.001 | KEGG |
|  | GLUCURONIDATION | -1.68 | 0.034 | WP698 |
|  | GLYCOSAMINOGLYCAN.METABOLISM | -1.63 | 0.044 | WP2743 |
|  | GLYCOSAMINOGLYCAN.DEGRADATION | -1.72 | 0.028 | KEGG |
|  | INTEGRIN.MEDIATED.CELL.ADHESION | -1.70 | 0.029 | WP185 |
|  | OTHER.GLYCAN.DEGRADATION | -2.06 | 0.002 | KEGG |
|  | MEMBRANE.TRAFFICKING | -2.67 | <0.001 | WP1846 |
|  | N.GLYCAN.BIOSYNTHESIS | -2.18 | 0.001 | KEGG |
|  | PDGF.PATHWAY | -1.87 | 0.009 | WP2526 |
|  | PI.METABOLISM | -1.75 | 0.023 | WP2747 |
|  | POST.TRANSLATIONAL.MODIFICATION.SYNTHESIS.OF.GPI.ANCHORED.PROTEINS | -1.69 | 0.032 | WP1887 |
|  | GLYCOSYLPHOSPHATIDYLINOSITOL.GPI.ANCHOR.BIOSYNTHESIS | -1.66 | 0.037 | KEGG |
|  | PROSTAGLANDIN.SYNTHESIS.AND.REGULATION | -1.64 | 0.042 | WP98 |
|  | REGULATION.OF.ACTIN.CYTOSKELETON | -1.79 | 0.018 | WP51 |
|  | REGULATION.OF.ACTIN.CYTOSKELETON | -1.97 | 0.004 | KEGG |
|  | CDC42RAC | -2.16 | 0.001 | BIOC |
|  | REGULATION.OF.MICROTUBULE.CYTOSKELETON | -1.74 | 0.024 | WP2038 |
|  | SIGNALING.BY.RHO.GTPASES | -1.74 | 0.024 | WP1917 |
|  | RHO | -2.03 | 0.003 | BIOC |
|  | SIGNALING.BY.ROBO.RECEPTOR | -1.74 | 0.024 | WP1918 |
|  | SPHINGOLIPID.METABOLISM | -2.28 | <0.001 | WP2788 |
|  | ADHERENS.JUNCTION | -2.28 | <0.001 | KEGG |
|  | UCALPAIN | -1.65 | 0.039 | BIOC |
|  | ENDOCYTOSIS | -1.95 | 0.005 | KEGG |
|  | LYSOSOME | -3.58 | <0.001 | KEGG |
|  | PEROXISOME | -1.76 | 0.021 | KEGG |
|  | UBIQUINONE.AND.OTHER.TERPENOID.QUINONE.BIOSYNTHESIS | -1.67 | 0.034 | KEGG |
|  | ECM | -1.88 | 0.009 | BIOC |
|  | GPCR | -1.64 | 0.040 | BIOC |
| Transcription & Translation | | | | |
|  | ACTIVATION.OF.CHAPERONE.GENES.BY.XBP1.S. | -2.09 | 0.001 | WP2667 |
|  | ACTIVATION.OF.GENE.EXPRESSION.BY.SREBP.SREBF. | -1.75 | 0.022 | WP2706 |
|  | CYTOPLASMIC.RIBOSOMAL.PROTEINS | -1.93 | 0.006 | WP477 |
|  | RIBOSOME | -2.39 | <0.001 | KEGG |
|  | DEADENYLATION.DEPENDENT.MRNA.DECAY | -2.14 | 0.001 | WP2659 |
|  | RIBOSOME.BIOGENESIS.IN.EUKARYOTES | -2.03 | 0.002 | KEGG |
|  | GENERIC.TRANSCRIPTION.PATHWAY | -1.95 | 0.005 | WP1822 |
|  | HISTONE.MODIFICATIONS | -2.42 | <0.001 | WP2369 |
|  | METABOLISM.OF.NON.CODING.RNA | -2.10 | 0.001 | WP2715 |
|  | METHYLATION.PATHWAYS | -1.61 | 0.049 | WP704 |
|  | MRNA.CAPPING | -2.22 | <0.001 | WP1861 |
|  | RNA.DEGRADATION | -2.39 | <0.001 | KEGG |
|  | MRNA.PROCESSING | -2.14 | 0.001 | WP411 |
|  | RNA.TRANSPORT | -2.55 | <0.001 | KEGG |
|  | NONSENSE.MEDIATED.DECAY | -1.97 | 0.004 | WP2710 |
|  | NUCLEOTIDE.EXCISION.REPAIR | -2.05 | 0.002 | WP1980 |
|  | NUCLEOTIDE.METABOLISM | -1.84 | 0.012 | WP404 |
|  | PROCESSING.OF.CAPPED.INTRON.CONTAINING.PRE.MRNA | -2.51 | <0.001 | WP1889 |
|  | SPLICEOSOME | -2.19 | <0.001 | KEGG |
|  | REGULATION.OF.DNA.REPLICATION | -2.81 | <0.001 | WP1898 |
|  | REGULATION.OF.MRNA.STABILITY.BY.PROTEINS.THAT.BIND.AU.RICH.ELEMENTS | -3.00 | <0.001 | WP2733 |
|  | REGULATORY.RNA.PATHWAYS | -2.30 | <0.001 | WP1901 |
|  | MRNA.SURVEILLANCE.PATHWAY | -2.25 | <0.001 | KEGG |
|  | RNA.POLYMERASE.I.RNA.POLYMERASE.III.AND.MITOCHONDRIAL.TRANSCRIPTION | -1.99 | 0.003 | WP1905 |
|  | RNA.POLYMERASE | -1.68 | 0.033 | KEGG |
|  | RNA.POLYMERASE.II.TRANSCRIPTION | -2.46 | <0.001 | WP1906 |
|  | RARRXR | -1.88 | 0.009 | BIOC |
|  | SIGNAL.REGULATORY.PROTEIN.SIRP.FAMILY.INTERACTIONS. | -1.61 | 0.049 | WP1909 |
|  | SRP.DEPENDENT.COTRANSLATIONAL.PROTEIN.TARGETING.TO.MEMBRANE | -2.36 | <0.001 | WP2737 |
|  | SYNTHESIS.OF.DNA | -2.68 | <0.001 | WP1925 |
|  | TRANSCRIPTIONAL.ACTIVATION.BY.NRF2 | -1.68 | 0.033 | WP3 |
|  | TRANSCRIPTIONAL.ACTIVITY.OF.SMAD2.SMAD3.SMAD4.HETEROTRIMER | -1.79 | 0.018 | WP2755 |
|  | TRANSCRIPTIONAL.REGULATION.OF.WHITE.ADIPOCYTE.DIFFERENTIATION | -2.23 | <0.001 | WP2751 |
|  | PPAR.SIGNALING.PATHWAY | -1.64 | 0.040 | KEGG |
|  | PPARA | -1.69 | 0.032 | BIOC |
|  | PPARG | -1.68 | 0.034 | BIOC |
|  | TRANSLATION.FACTORS | -1.77 | 0.020 | WP107 |
|  | TRNA.AMINOACYLATION | -2.09 | 0.001 | WP1938 |
|  | AMINOACYL.TRNA.BIOSYNTHESIS | -1.90 | 0.008 | KEGG |
|  | WNT.LIGAND.BIOGENESIS.AND.TRAFFICKING | -1.69 | 0.032 | WP2790 |
|  | WNT.SIGNALING.PATHWAY.NETPATH | -1.62 | 0.045 | WP363 |
|  | WNT | -1.62 | 0.046 | BIOC |
|  | BASAL.TRANSCRIPTION.FACTORS | -1.95 | 0.005 | KEGG |
|  | CARM_ER | -1.67 | 0.036 | BIOC |
|  | CARM1 | -1.66 | 0.037 | BIOC |
| Metabolism mitochondria | | | | |
|  | ACTIVATION.OF.CHAPERONE.GENES.BY.ATF6.ALPHA | -1.77 | 0.020 | WP2655 |
|  | ACTIVATION.OF.GENES.BY.ATF4 | -1.82 | 0.014 | WP2753 |
|  | ELECTRON.TRANSPORT.CHAIN | -2.81 | <0.001 | WP111 |
|  | ETC | -1.81 | 0.015 | BIOC |
|  | ENERGY.METABOLISM | -2.29 | <0.001 | WP1541 |
|  | MITOCHONDRIAL.PROTEIN.IMPORT | -1.86 | 0.010 | WP2717 |
|  | NOTCH.SIGNALING.PATHWAY | -2.14 | 0.001 | WP61 |
|  | NOTCH.SIGNALING.PATHWAY | -1.96 | 0.005 | WP268 |
|  | OXIDATIVE.PHOSPHORYLATION | -2.36 | <0.001 | WP623 |
|  | OXIDATIVE.PHOSPHORYLATION | -2.79 | <0.001 | KEGG |
|  | PRE.NOTCH.EXPRESSION.AND.PROCESSING | -1.82 | 0.014 | WP2786 |
|  | RESPIRATORY.ELECTRON.TRANSPORT.ATP.SYNTHESIS.BY.CHEMIOSMOTIC.COUPLING.AND.HEAT.PRODUCTION.BY.UNCOUPLING.PROTEINS. | -2.58 | <0.001 | WP1902 |
|  | SIGNALING.BY.NOTCH1 | -2.18 | 0.001 | WP2720 |
|  | SIGNALING.BY.NOTCH2 | -2.03 | 0.002 | WP2718 |
|  | SIGNALING.BY.NOTCH3 | -1.65 | 0.038 | WP2722 |
|  | NOTCH.SIGNALING.PATHWAY | -2.11 | 0.001 | KEGG |
|  | TCA.CYCLE | -2.22 | <0.001 | WP78 |
|  | CITRATE.CYCLE.TCA.CYCLE. | -2.24 | <0.001 | KEGG |
|  | KREB | -1.65 | 0.039 | BIOC |
|  | THE.CITRIC.ACID.TCA.CYCLE.AND.RESPIRATORY.ELECTRON.TRANSPORT. | -2.61 | <0.001 | WP2766 |
|  | MALATEX | -1.67 | 0.036 | BIOC |
| Metabolism Protein/AA | | | | |
|  | DEGRADATION.OF.BETA.CATENIN.BY.THE.DESTRUCTION.COMPLEX | -2.72 | <0.001 | WP2773 |
|  | METABOLISM.OF.AMINO.ACIDS.AND.DERIVATIVES | -2.34 | <0.001 | WP2693 |
|  | AMINO.SUGAR.AND.NUCLEOTIDE.SUGAR.METABOLISM | -1.89 | 0.008 | KEGG |
|  | METABOLISM.OF.NITRIC.OXIDE | -1.79 | 0.018 | WP1850 |
|  | PARKIN.UBIQUITIN.PROTEASOMAL.SYSTEM.PATHWAY | -1.83 | 0.014 | WP2359 |
|  | UBIQUITIN.MEDIATED.PROTEOLYSIS | -2.39 | <0.001 | KEGG |
|  | PROTEASOME.DEGRADATION | -2.78 | <0.001 | WP183 |
|  | PROTEASOME | -2.34 | <0.001 | KEGG |
|  | PROTEASOME | -2.42 | <0.001 | BIOC |
|  | PROTEIN.FOLDING | -1.84 | 0.012 | WP1892 |
|  | PROTEIN.PROCESSING.IN.ENDOPLASMIC.RETICULUM | -3.09 | <0.001 | KEGG |
|  | VALINE.LEUCINE.AND.ISOLEUCINE.DEGRADATION | -2.15 | 0.001 | KEGG |
|  | LYSINE.DEGRADATION | -2.14 | 0.001 | KEGG |
|  | PROTEIN.EXPORT | -2.37 | <0.001 | KEGG |
| Metabolism substrate & energy | | | | |
|  | AMPK.SIGNALING | -1.76 | 0.022 | WP1403 |
|  | AMPK.SIGNALING.PATHWAY | -2.17 | 0.001 | KEGG |
|  | CORI.CYCLE | -1.89 | 0.008 | WP1946 |
|  | PYRUVATE.METABOLISM | -2.21 | <0.001 | KEGG |
|  | ENERGY.DEPENDENT.REGULATION.OF.MTOR.BY.LKB1.AMPK | -1.68 | 0.033 | WP2748 |
|  | MTOR.SIGNALING.PATHWAY | -1.80 | 0.017 | KEGG |
|  | FATTY.ACID.BETA.OXIDATION | -2.05 | 0.002 | WP143 |
|  | FATTY.ACID.DEGRADATION | -1.89 | 0.008 | KEGG |
|  | FATTY.ACID.BIOSYNTHESIS | -2.31 | <0.001 | WP357 |
|  | FATTY.ACID.ELONGATION | -2.19 | <0.001 | KEGG |
|  | FATTY.ACID.TRIACYLGLYCEROL.AND.KETONE.BODY.METABOLISM | -2.25 | <0.001 | WP1817 |
|  | GLYCEROPHOSPHOLIPID.BIOSYNTHESIS | -1.72 | 0.027 | WP2740 |
|  | GLYCOLYSIS.AND.GLUCONEOGENESIS | -1.78 | 0.019 | WP534 |
|  | HEXOSE.TRANSPORT | -2.00 | 0.003 | WP1828 |
|  | INSULIN.PROCESSING | -1.67 | 0.035 | WP2736 |
|  | INSULIN.SIGNALING | -1.66 | 0.037 | WP481 |
|  | INSULIN.SIGNALING.PATHWAY | -1.69 | 0.032 | KEGG |
|  | MAPK.CASCADE | -1.78 | 0.018 | WP422 |
|  | MAPK.SIGNALING.PATHWAY | -2.08 | 0.002 | WP382 |
|  | METABOLISM.OF.CARBOHYDRATES | -2.08 | 0.001 | WP1848 |
|  | MITOCHONDRIAL.LC.FATTY.ACID.BETA.OXIDATION | -1.81 | 0.015 | WP368 |
|  | PENTOSE.PHOSPHATE.PATHWAY | -1.85 | 0.011 | WP134 |
|  | PENTOSE.PHOSPHATE.PATHWAY | -2.23 | <0.001 | KEGG |
|  | REGULATION.OF.LIPID.METABOLISM.BY.PEROXISOME.PROLIFERATOR.ACTIVATED.RECEPTOR.ALPHA.PPARALPHA. | -2.24 | <0.001 | WP2797 |
|  | SIGNALING.BY.INSULIN.RECEPTOR | -1.89 | 0.008 | WP1913 |
|  | TRANSLOCATION.OF.GLUT4.TO.THE.PLASMA.MEMBRANE | -2.31 | <0.001 | WP2777 |
|  | BIOSYNTHESIS.OF.UNSATURATED.FATTY.ACIDS | -2.04 | 0.002 | KEGG |
|  | GALACTOSE.METABOLISM | -1.65 | 0.039 | KEGG |
|  | STARCH.AND.SUCROSE.METABOLISM | -1.81 | 0.015 | KEGG |
| Cholesterol & Micronutrients | | | | |
|  | CHOLESTEROL.BIOSYNTHESIS | -1.71 | 0.028 | WP197 |
|  | IRON.UPTAKE.AND.TRANSPORT | -2.15 | 0.001 | WP2670 |
|  | METABOLISM.OF.WATER.SOLUBLE.VITAMINS.AND.COFACTORS | -1.62 | 0.046 | WP1857 |
|  | MINERAL.ABSORPTION | -1.68 | 0.034 | KEGG |
|  | REGULATION.OF.CHOLESTEROL.BIOSYNTHESIS.BY.SREBP.SREBF. | -2.10 | 0.001 | WP2686 |
|  | TERPENOID.BACKBONE.BIOSYNTHESIS | -2.11 | 0.001 | KEGG |
|  | SELENIUM.METABOLISM.AND.SELENOPROTEINS | -1.68 | 0.034 | WP28 |
|  | SREBP.SIGNALLING | -2.23 | <0.001 | WP1982 |
|  | SULFUR.METABOLISM | -1.75 | 0.023 | KEGG |
| Endocrine function | | | | |
|  | LEPTIN.SIGNALING.PATHWAY | -1.75 | 0.022 | WP2034 |
|  | SEROTONIN.HTR1.GROUP.AND.FOS.PATHWAY | -1.74 | 0.024 | WP722 |
| oxidative stress | | | | |
|  | DETOXIFICATION.OF.REACTIVE.OXYGEN.SPECIES | -2.22 | <0.001 | WP2824 |
|  | FOXO.SIGNALING.PATHWAY | -2.14 | 0.001 | KEGG |
|  | FAS.PATHWAY.AND.STRESS.INDUCTION.OF.HSP.REGULATION | -1.79 | 0.018 | WP314 |
|  | GLUTATHIONE.METABOLISM | -1.61 | 0.048 | WP100 |
|  | GLUTATHIONE.METABOLISM | -2.18 | 0.001 | KEGG |
|  | OXIDATIVE.STRESS | -2.12 | 0.001 | WP408 |
|  | UNFOLDED.PROTEIN.RESPONSE | -1.73 | 0.025 | WP1939 |
|  | TRANS.SULFURATION.AND.ONE.CARBON.METABOLISM | -1.71 | 0.028 | WP2525 |
|  | TOR.SIGNALING | -1.98 | 0.004 | WP1471 |
|  | HIF.1.SIGNALING.PATHWAY | -1.69 | 0.032 | KEGG |
| Inflammation | | | | |
|  | EBV.LMP1.SIGNALING | -1.69 | 0.032 | WP262 |
|  | FC.EPSILON.RECEPTOR.FCERI.SIGNALING. | -2.02 | 0.003 | WP2759 |
|  | IL.1.SIGNALING.PATHWAY | -1.95 | 0.005 | WP195 |
|  | IL.5.SIGNALING.PATHWAY | -1.64 | 0.040 | WP127 |
|  | IL.6.SIGNALING.PATHWAY | -1.92 | 0.006 | WP364 |
|  | IL.7.SIGNALING.PATHWAY | -1.77 | 0.020 | WP205 |
|  | IL.9.SIGNALING.PATHWAY | -1.93 | 0.006 | WP22 |
|  | INTERFERON.ALPHA.BETA.SIGNALING | -1.87 | 0.010 | WP1835 |
|  | IFNA | -1.97 | 0.004 | BIOC |
|  | INTERFERON.GAMMA.SIGNALING | -2.39 | <0.001 | WP1836 |
|  | IFNG | -1.76 | 0.021 | BIOC |
|  | INTERFERON.TYPE.I.SIGNALING.PATHWAYS | -1.89 | 0.009 | WP585 |
|  | IL10 | -1.84 | 0.012 | BIOC |
|  | INTERLEUKIN.11.SIGNALING.PATHWAY | -1.72 | 0.028 | WP2332 |
|  | MYD88.CASCADE.INITIATED.ON.PLASMA.MEMBRANE | -1.84 | 0.012 | WP2801 |
|  | MYD88.DEPENDENT.CASCADE.INITIATED.ON.ENDOSOME | -1.82 | 0.014 | WP2768 |
|  | MYD88.MAL.CASCADE.INITIATED.ON.PLASMA.MEMBRANE | -2.28 | <0.001 | WP2761 |
|  | RANKL.RANK.SIGNALING.PATHWAY | -1.64 | 0.041 | WP2018 |
|  | REGULATION.OF.TOLL.LIKE.RECEPTOR.SIGNALING.PATHWAY | -1.92 | 0.006 | WP1449 |
|  | RESPONSE.TO.ELEVATED.PLATELET.CYTOSOLIC.CA2. | -1.78 | 0.018 | WP1903 |
|  | STRUCTURAL.PATHWAY.OF.INTERLEUKIN.1.IL.1. | -1.92 | 0.006 | WP2637 |
|  | TNF.ALPHA.SIGNALING.PATHWAY | -2.18 | <0.001 | WP2808 |
|  | TNF.SIGNALING.PATHWAY | -1.70 | 0.030 | KEGG |
|  | TNF.ALPHA.SIGNALING.PATHWAY | -2.13 | 0.001 | WP231 |
|  | TOLL.LIKE.RECEPTOR.SIGNALING.PATHWAY | -2.26 | <0.001 | WP75 |
|  | TOLL.LIKE.RECEPTOR.SIGNALING.PATHWAY | -2.24 | <0.001 | KEGG |
|  | TOLL | -1.69 | 0.031 | BIOC |
|  | TOLL.LIKE.RECEPTORS.CASCADES | -2.05 | 0.002 | WP2775 |
|  | TCRA | -1.64 | 0.041 | BIOC |
|  | TSH.SIGNALING.PATHWAY | -1.78 | 0.019 | WP2032 |
|  | THYROID.HORMONE.SIGNALING.PATHWAY | -2.05 | 0.002 | KEGG |
|  | TYPE.II.INTERFERON.SIGNALING.IFNG. | -2.31 | <0.001 | WP619 |
|  | NOD.LIKE.RECEPTOR.SIGNALING.PATHWAY | -1.72 | 0.027 | KEGG |
|  | NF.KAPPA.B.SIGNALING.PATHWAY | -1.66 | 0.037 | KEGG |
|  | NFKB | -1.62 | 0.046 | BIOC |
| Autophagy/apoptosis | | | | |
|  | APOPTOSIS.MODULATION.BY.HSP70 | -1.89 | 0.008 | WP384 |
|  | APOPTOTIC.EXECUTION.PHASE | -1.74 | 0.024 | WP1784 |
|  | REGULATION.OF.APOPTOSIS | -2.62 | <0.001 | WP1896 |
|  | SENESCENCE.AND.AUTOPHAGY | -2.01 | 0.003 | WP615 |
|  | REGULATION.OF.AUTOPHAGY | -1.84 | 0.012 | KEGG |
|  | SIGNALING.BY.EGFR | -2.48 | <0.001 | WP1910 |
|  | SIGNALLING.BY.NGF | -1.89 | 0.008 | WP1976 |
| Immune system | | | | |
|  | ADVANCED.GLYCOSYLATION.ENDPRODUCT.RECEPTOR.SIGNALING | -1.74 | 0.024 | WP1781 |
|  | AGE.RAGE.PATHWAY | -2.11 | 0.001 | WP2324 |
|  | AMYLOIDS | -2.06 | 0.002 | WP2739 |
|  | ASPARAGINE.N.LINKED.GLYCOSYLATION | -2.69 | <0.001 | WP1785 |
|  | B.CELL.RECEPTOR.SIGNALING.PATHWAY | -2.03 | 0.002 | WP23 |
|  | B.CELL.RECEPTOR.SIGNALING.PATHWAY | -1.80 | 0.017 | KEGG |
|  | BLYMPHOCYTE | -1.75 | 0.023 | BIOC |
|  | CLASS.I.MHC.MEDIATED.ANTIGEN.PROCESSING.AMP.PRESENTATION | -3.07 | <0.001 | WP2796 |
|  | ANTIGEN.PROCESSING.AND.PRESENTATION | -2.79 | <0.001 | KEGG |
|  | COSTIMULATION.BY.THE.CD28.FAMILY | -2.13 | 0.001 | WP1799 |
|  | HUMAN.COMPLEMENT.SYSTEM | -1.69 | 0.032 | WP2806 |
|  | MHC.CLASS.II.ANTIGEN.PRESENTATION | -2.81 | <0.001 | WP2679 |
|  | SIGNALING.BY.THE.B.CELL.RECEPTOR.BCR. | -2.49 | <0.001 | WP2746 |
|  | SIGNALING.BY.TGF.BETA.RECEPTOR.COMPLEX | -1.83 | 0.013 | WP2742 |
|  | SIGNALING.BY.ERBB2 | -1.61 | 0.047 | WP2780 |
|  | SIGNALING.BY.ERBB4 | -1.96 | 0.005 | WP2781 |
|  | TCR.SIGNALING | -2.30 | <0.001 | WP1927 |
|  | CSK | -1.70 | 0.031 | BIOC |
|  | TCR.SIGNALING.PATHWAY | -1.86 | 0.010 | WP69 |
|  | T.CELL.RECEPTOR.SIGNALING.PATHWAY | -1.79 | 0.018 | KEGG |
|  | CTLA4 | -1.67 | 0.035 | BIOC |
|  | NKCELLS | -1.72 | 0.026 | BIOC |
|  | TH1TH2 | -1.78 | 0.019 | BIOC |
|  | THELPER | -1.69 | 0.031 | BIOC |
|  | MEF2D | -1.98 | 0.004 | BIOC |
|  | FCER1 | -1.95 | 0.005 | BIOC |
| Infection, disease, cancer | | | | |
|  | ACUTE.MYELOID.LEUKEMIA | -1.64 | 0.042 | KEGG |
|  | AMI | -1.76 | 0.021 | BIOC |
|  | ALCOHOLISM | -1.69 | 0.032 | KEGG |
|  | ALLOGRAFT.REJECTION | -2.33 | <0.001 | KEGG |
|  | ALZHEIMERS.DISEASE | -1.79 | 0.017 | WP2059 |
|  | ALZHEIMER.S.DISEASE | -2.34 | <0.001 | KEGG |
|  | ASTHMA | -2.11 | 0.001 | KEGG |
|  | AUTOIMMUNE.THYROID.DISEASE | -2.16 | 0.001 | KEGG |
|  | BACTERIAL.INVASION.OF.EPITHELIAL.CELLS | -1.85 | 0.011 | KEGG |
|  | BLADDER.CANCER | -1.72 | 0.027 | KEGG |
|  | CHAGAS.DISEASE.AMERICAN.TRYPANOSOMIASIS. | -2.10 | 0.001 | KEGG |
|  | CHRONIC.MYELOID.LEUKEMIA | -2.07 | 0.002 | KEGG |
|  | ENDOMETRIAL.CANCER | -1.67 | 0.035 | KEGG |
|  | EPITHELIAL.CELL.SIGNALING.IN.HELICOBACTER.PYLORI.INFECTION | -2.29 | <0.001 | KEGG |
|  | EPSTEIN.BARR.VIRUS.INFECTION | -2.69 | <0.001 | KEGG |
|  | GASTRIC.CANCER.NETWORK.1 | -1.66 | 0.038 | WP2361 |
|  | GLIOMA | -1.62 | 0.045 | KEGG |
|  | SIGNALING.PATHWAYS.IN.GLIOBLASTOMA | -2.14 | 0.001 | WP2261 |
|  | GRAFT.VERSUS.HOST.DISEASE | -2.37 | <0.001 | KEGG |
|  | HEPATITIS.B | -2.31 | <0.001 | KEGG |
|  | HEPATITIS.C | -2.22 | <0.001 | KEGG |
|  | HERPES.SIMPLEX.INFECTION | -2.92 | <0.001 | KEGG |
|  | HCMV | -1.85 | 0.011 | BIOC |
|  | HIV.LIFE.CYCLE | -2.93 | <0.001 | WP2658 |
|  | HIVNEF | -2.05 | 0.002 | BIOC |
|  | HOST.INTERACTIONS.OF.HIV.FACTORS | -3.02 | <0.001 | WP2684 |
|  | HTLV.I.INFECTION | -1.83 | 0.013 | KEGG |
|  | HUNTINGTON.S.DISEASE | -2.56 | <0.001 | KEGG |
|  | INFLAMMATORY.BOWEL.DISEASE.IBD. | -2.16 | 0.001 | KEGG |
|  | INFLUENZA.A | -2.97 | <0.001 | KEGG |
|  | INFLUENZA.LIFE.CYCLE | -2.47 | <0.001 | WP2683 |
|  | NTHI | -1.72 | 0.028 | BIOC |
|  | INTEGRATED.BREAST.CANCER.PATHWAY | -2.33 | <0.001 | WP1984 |
|  | ATRBRCA | -1.64 | 0.042 | BIOC |
|  | INTEGRATED.CANCER.PATHWAY | -1.91 | 0.007 | WP1971 |
|  | INTEGRATED.PANCREATIC.CANCER.PATHWAY | -1.66 | 0.037 | WP2377 |
|  | ISG15.ANTIVIRAL.MECHANISM | -2.04 | 0.002 | WP2672 |
|  | LATENT.INFECTION.OF.HOMO.SAPIENS.WITH.MYCOBACTERIUM.TUBERCULOSIS | -2.24 | <0.001 | WP2700 |
|  | LEGIONELLOSIS | -2.30 | <0.001 | KEGG |
|  | LEISHMANIASIS | -2.36 | <0.001 | KEGG |
|  | LONG.TERM.DEPRESSION | -1.83 | 0.013 | KEGG |
|  | MEASLES | -2.79 | <0.001 | KEGG |
|  | NON.ALCOHOLIC.FATTY.LIVER.DISEASE.NAFLD. | -2.12 | 0.001 | KEGG |
|  | NON.SMALL.CELL.LUNG.CANCER | -1.65 | 0.039 | KEGG |
|  | PANCREATIC.CANCER | -2.06 | 0.002 | KEGG |
|  | PARKINSON.S.DISEASE | -2.33 | <0.001 | KEGG |
|  | PATHOGENIC.ESCHERICHIA.COLI.INFECTION | -1.93 | 0.006 | WP2272 |
|  | PATHOGENIC.ESCHERICHIA.COLI.INFECTION | -2.03 | 0.003 | KEGG |
|  | PERTUSSIS | -1.80 | 0.016 | KEGG |
|  | PROSTATE.CANCER | -1.76 | 0.021 | WP2263 |
|  | PROSTATE.CANCER | -1.90 | 0.008 | KEGG |
|  | PROTEOGLYCANS.IN.CANCER | -1.74 | 0.023 | KEGG |
|  | RB.IN.CANCER | -1.91 | 0.007 | WP2446 |
|  | RENAL.CELL.CARCINOMA | -1.73 | 0.026 | KEGG |
|  | RHEUMATOID.ARTHRITIS | -2.53 | <0.001 | KEGG |
|  | SALMONELLA.INFECTION | -2.19 | <0.001 | KEGG |
|  | SALMONELLA | -1.87 | 0.010 | BIOC |
|  | SHIGELLOSIS | -1.94 | 0.006 | KEGG |
|  | STAPHYLOCOCCUS.AUREUS.INFECTION | -2.54 | <0.001 | KEGG |
|  | SYSTEMIC.LUPUS.ERYTHEMATOSUS | -2.68 | <0.001 | KEGG |
|  | TGF.BETA.SIGNALING.PATHWAY | -1.76 | 0.022 | KEGG |
|  | TOXOPLASMOSIS | -2.52 | <0.001 | KEGG |
|  | TRANSCRIPTIONAL.MISREGULATION.IN.CANCER | -1.85 | 0.011 | KEGG |
|  | TUBERCULOSIS | -2.68 | <0.001 | KEGG |
|  | TYPE.I.DIABETES.MELLITUS | -2.17 | 0.001 | KEGG |
|  | VIBRIO.CHOLERAE.INFECTION | -2.65 | <0.001 | KEGG |
|  | VIRAL.CARCINOGENESIS | -2.60 | <0.001 | KEGG |
|  | VIRAL.MYOCARDITIS | -2.67 | <0.001 | KEGG |

NES, normalized enrichment score; q, statistical significance of false discovery rate (FDR) of post versus pre-enrichment comparing changes after E+R versus changes after placebo; Source represents the database, in which the respective pathway has been identified, KEGG, Kyoto Encyclopedia of Genes; and Genomes, WP, Wikipathways, and BIOC, Biocarta.

Supplementary Table S3. Ingenuity upstream analysis including related genes

|  | Upstream regulator | Downstream targets/pathways |  | genes | P |
| --- | --- | --- | --- | --- | --- |
| Adipogenesis |  |  |  |  |  |
| Inhibited | β-Estradiol | adipogenesis | chemical - endogenous mammalian | 49 | 0.040 |
| Inhibited | Prolactin | early in adipogenesis | cytokine | 30 | <0.001 |
| Activated | miR-124-3p | adipogenic | mature microRNA | 24 | 0.001 |
|  |  |  |  |  |  |
| Oxidative stress |  |  |  |  |  |
| Inhibited | Genistein | PPARs, NRF, autophagy | chemical drug | 34 | <0.001 |
| Inhibited | NFE2L2, =NRF2 | antioxidant defense | transcription regulator | 31 | 0.001 |
|  |  |  |  |  |  |
| Inflammation and Immune defense | | |  |  |  |
| Inhibited | TNF | inflammation | cytokine | 49 | 0.023 |
| Activated | Sirolimus (=Rapamycin) | impairs T- & B-cell proliferation and activation | chemical drug | 29 | 0.007 |
|  |  |  |  |  |  |
| Anti-carcinogenic |  |  |  |  |  |
| Inhibited | Trichostatin A | antibiotic, inhibits histone deacetylase | chemical drug | 30 | 0.040 |
| Inhibited | Gentamicin | antibiotic, binds bacterial ribosome | chemical drug | 24 | 0.002 |
| Activated | 5-Fluorouracil | cytostaticum | chemical drug | 26 | <0.001 |
| Activated | CD 437 | adipogenesis, anti-carcinogenic (by ER-stress) | chemical drug | 25 | <0.001 |

PPAR-α, Peroxisome proliferator-activated receptor α; NFE2L2, Nuclear factor (erythroid-derived 2)-like 2 (=NRF2); TNF-α, tumor necrosis factor α. P, statistical significance of activation/inhibition of upstream regulator for post versus pre-enrichment.

Supplementary Table S4. Primer Sequences

| Gene | Primer sequence |
| --- | --- |
| ATP6V1A |  |
| F | GAGATCCTGTACTTCGCACTGG |
| R | GGGGATGTAGATGCTTTGGGT |
| ATP6V1H |  |
| F | CAGAAGTTCGTGCAAACAAAGTC |
| R | TCAGGGCTTCGTTTCATTTCAA |
| CD68 |  |
| F | CCCTATGGACACCTCAGCTTT |
| R | GAAGGACACATTGTACTCCACC |
| GAPDH |  |
| F | GAGTCAACGGATTTGGTCGT |
| R | TTGATTTTGGAGGGATCTCG |
| HSL (LIPE) |  |
| F | GCGGATCACACAGAACCTGG AC |
| R | AGCAGGCGGCTTACCCT CAC |
| LAMP2 |  |
| F | GAAAATGCCACTTGCCTTTATGC |
| R | AGGAAAAGCCAGGTCCGAAC |
| PI4K2A |  |
| F | TTGTCCTTAACCAGGGCTATCT |
| R | GTACGGGGAACAATGTTGAGT |
| UCP2 |  |
| F | GGAGGTGGTCGGAGATACCAA |
| R | ACAATGGCATTACGAGCAACAT |

Primer sequences for SYBR-Green based RT-qPCR analyses. F, forward; R, reverse.
